# M2 macrophage-derived exosomal microRNAs inhibit cell migration and invasion in gliomas through PI3K/AKT/mTOR signaling pathway

**DOI:** 10.1186/s12967-021-02766-w

**Published:** 2021-03-06

**Authors:** Jie Yao, Zefen Wang, Yong Cheng, Chao Ma, Yahua Zhong, Yilei Xiao, Xu Gao, Zhiqiang Li

**Affiliations:** 1 Human Genetic Resources Conservation Center of Hubei Province, Wuhan, 430071 China; 2 Tumor Precision Diagnosis and Treatment Technology and Translation Medicine, Hubei Engineering Research Center, Wuhan, 430071 China; 3 Department of Physiology, Wuhan University School of Basic Medical Sciences, Wuhan, 430071 China; 4 Department of Neurology, Hankou Hospital, General Hospital of Central Theater Command of Chinese People’s Liberation Army, Wuhan, 430014 China; 5 Department of Neurosurgery, Zhongnan Hospital of Wuhan University, No 169 Donghu Road, Wuhan, 430071 Hubei China; 6 Department of Oncology, Zhongnan Hospital of Wuhan University, Wuhan, 430071 China; 7 Department of Neurosurgery, Liaocheng People’s Hospital, Liaocheng, 252000 China; 8 Department of Neurosurgery, General Hospital of Northern Theater Command of People’s Liberation Army, Shenyang, 110000 China

**Keywords:** M2 macrophage, Exosome, hsa-miR-15a-5p, hsa-miR-92a-3p, Glioma, PI3K/AKT/mTOR

## Abstract

**Background:**

Glioma, the most common primary brain tumor, account Preparing figures for 30 to 40% of all intracranial tumors. Herein, we aimed to study the effects of M2 macrophage-derived exosomal microRNAs (miRNAs) on glioma cells.

**Methods:**

First, we identified seven differentially expressed miRNAs in infiltrating macrophages and detected the expression of these seven miRNAs in M2 macrophages. We then selected hsa-miR-15a-5p (miR-15a) and hsa-miR-92a-3p (miR-92a) for follow-up studies, and confirmed that miR-15a and miR-92a were under-expressed in M2 macrophage exosomes. Subsequently, we demonstrated that M2 macrophage-derived exosomes promoted migration and invasion of glioma cells, while exosomal miR-15a and miR-92a had the opposite effects on glioma cells. Next, we performed the target gene prediction in four databases and conducted target gene validation by qRT-PCR, western blot and dual luciferase reporter gene assays.

**Results:**

The results revealed that miR-15a and miR-92a were bound to CCND1 and RAP1B, respectively. Western blot assays demonstrated that interference with the expression of CCND1 or RAP1B reduced the phosphorylation level of AKT and mTOR, indicating that both CCND1 and RAP1B can activate the PI3K/AKT/mTOR signaling pathway.

**Conclusion:**

Collectively, these findings indicate that M2 macrophage-derived exosomal miR-15a and miR-92a inhibit cell migration and invasion of glioma cells through PI3K/AKT/mTOR signaling pathway.

**Supplementary information:**

The online version contains supplementary material available at 10.1186/s12967-021-02766-w.

## Introduction

Gliomas, one of the most common types of primary brain tumors, arise from the gluey supportive cells that surround nerve cells and help them function [1, 2]. Despite an increased molecular understanding of gliomas and advances in treatment options including surgery, radiation therapy, chemotherapy, and targeted therapy, almost all (> 90%) malignant gliomas still recur, most commonly within 3 cm of the original tumor margin [3, 4]. Once a relapse is diagnosed, the survival time is short, usually 3–6 months [5]. Therefore, there is an urgent need to find potential biomarkers and effective treatment strategies to reduce malignant gliomas characterized by low survival rates.

It is well established that tumor microenvironment plays a critical role in promoting tumor growth and metastasis [6, 7]. Tumor-associated macrophages (TAM) are the most abundant myeloid cells infiltrating the tumor microenvironment [8, 9]. Activated macrophages are generally classified as M1/M2 dichotomy, which are two extremes in the functional state spectrum [10, 11]. Th1 cytokine-induced M1 macrophages have pro-inflammatory and antitumor activities, while Th2 cytokine-driven M2 macrophages increase angiogenesis and show tumor-promoting functions [12]. In recent studies, M2 macrophages have been proven to promote glioma proliferation and migration abilities [13, 14].

Exosomes, small membrane vesicles containing mRNAs, microRNAs (miRNAs), long noncoding RNAs and proteins, play a vital role in intercellular communication by delivering their contents to recipient cells [15, 16]. Lan et al. elucidated that M2 macrophages promote the migration and invasion of colorectal cancer cells by M2 macrophage-derived exosomes which show high expression levels of miR-21-5p and miR-155-5p [17]. Zheng et al. demonstrated that the exosome-mediated transfer of functional ApoE protein from M2 macrophages to the tumor cells promotes the migration of gastric cancer cells [18]. However, the role of M2 macrophage-derived exosomes in regulating glioma progression and metastasis remains largely unknown.

In the present study, differentially expressed miRNAs between peripheral blood mononuclear cells and infiltrating macrophages were identified and their expression levels were investigated in M2 macrophages and in exosomes derived from M2 macrophages. Next, we evaluated the effects of M2 macrophage-derived exosomes and exosomal hsa-miR-15a-5p (miR-15a) and hsa-miR-92a-3p (miR-92a) on migration and invasion of glioma cells. Subsequently, target gene prediction and validation of miR-15a and miR-92a were implemented. Finally, western blot analysis was performed to explore the relationship between the expression of target genes and PI3K/AKT/mTOR signaling pathway.

## Results

### Identification and verification of key miRNAs in M2 macrophage-derived exosomes

**Fig. 1:**
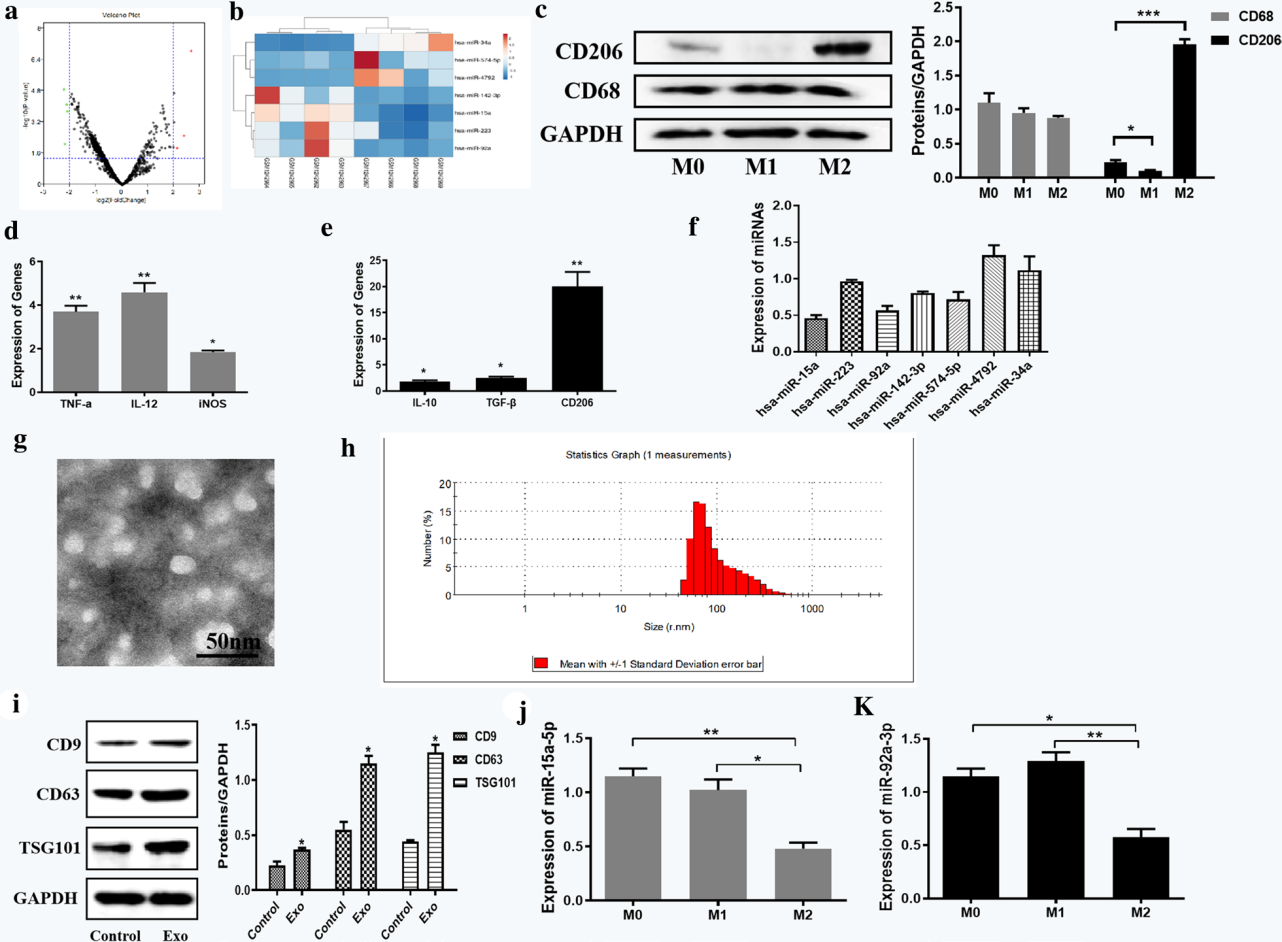
Identification and verification of key miRNAs in M2 macrophage-derived exosomes. **a** Volcano plot of dataset GSE51332. **b** Heat maps of the expression profiles of seven miRNAs. **c** Expression of CD206 and CD68 in M0, M1, M2 macrophages. **d** Biomarkers detection in M1 macrophages compared to M2 macrophages. **e** Biomarkers detection of M2 macrophages compared to M1 macrophages. **f** Relative expressions of selected seven miRNAs in M2 macrophages compared to M1 macrophages. **g** Identification of exosome structure under JEM-2010 HT transmission electron microscope (Scale bar = 50 nm). **h** The size distributions and numbers of exosomes was analyzed by NTA. **i** Biomarker detection of M2 macrophage exosomes. **j** Expression of miR-15a in exosomes derived from macrophages with different differentiation states. **k** Expression of miR-92a in exosomes derived from macrophages with different differentiation states. Error bars denote standard deviation of triplicates. ***P < 0.001; **P < 0.01; *P < 0.05

We obtained the dataset GSE51332 from GEO database, which included four cases of peripheral blood mononuclear cells and four cases of infiltrating macrophages in glioma patients. By analyzing the miRNA expression in the samples through GEO2R (Fig. 1a), we identified seven miRNAs that were most significantly differentially expressed in infiltrating macrophages (Table 1). By visualizing the expression in the samples through ClusterVis, we obtained the heat maps of the expression profiles of these seven miRNAs (Fig. 1b).

**Table 1:** Information of differentially expressed miRNAs

miRNA ID	Log_2_FC	*P* value
hsa-miR-15a	− 2.24935	1.27E−05
hsa-miR-223	− 2.21532	8.15E−03
hsa-miR-92a	− 2.14024	7.60E−05
hsa-miR-142-3p	− 2.11088	1.71E−04
hsa-miR-574-5p	2.10026	1.35E−02
hsa-miR-4792	2.36518	2.96E−03
hsa-miR-34a	2.65544	1.36E−07

Subsequently, we tested the expression levels of biomarkers in macrophages with different differentiation states. The results of western blot assays (Fig. 1c) showed that CD206 (M2 macrophage phenotype marker gene) was significantly over-expressed in M2 macrophages, and the expression of CD68 (macrophage marker gene) was not significantly different in M0, M1 and M2 macrophages. By qRT-PCR detection, we found that TNF-a, IL-12 and iNOS (M1 macrophage phenotype marker genes) were up-regulated in M1 macrophages compared with M0 macrophages (Fig. 1d). IL-10, TGF-β, and CD206 (M2 macrophage phenotype marker genes) were found to be highly expressed in M2 macrophages compared with M0 macrophages (Fig. 1e). Then we induced macrophage differentiation according to the tested markers through in vitro cell experiments and measured the expression of these seven miRNAs using qRT-PCR. The results elucidated that miR-15a and miR-92a were significantly under-expressed in M2 macrophages (Fig. 1f), consistent with the expression levels in GEO. However, no obvious upregulated expression levels were shown in the detection of miR-574, miR-4792 and miR-34a, which was not consistent with their expressed results predicted in GEO. Therefore, we selected the obviously downregulated miRNAs, miR-15a and miR-92a, as the next research objects.

In order to verify the expression of miR-15a and miR-92a in exosomes derived from M2 macrophages, exosomes need to be extracted from M2 macrophages. Thus, we collected the culture supernatant of M2 macrophages and THP-1 cells which were used as the control group. According to the TEM image obtained in JEM-2010 HT transmission electron microscope (Fig. 1g), the supernatant of M2 macrophage cell culture contained exosomes, of which the shape was solid and dense. Besides, analysis of size distribution of exosomes was assayed by NTA. As shown in Fig. 1h, the isolated exosomes had a predominant size of 70–120 nm. Exosomes were then extracted and the expression levels of CD9, CD63, and TSG101 (biomarkers of exosomes) were determined by western blot assays. The results showed that the protein levels of TSG101, CD63 and CD81 were significantly increased in the Exo group (Fig. 1i), while the protein levels of Calnexin was evidently diminish in the Exo group compared with control group (Additional file 1: Figure S1), which further confirmed the successful extraction of exosomes. Then we collected the culture supernatants of macrophages with different differentiation states, extracted the exosomes, and examined the expression of miR-15a and miR-92a. As shown in Fig. 1j and k, miR-15a and 92a were under-expressed in M2 macrophage exosomes, and their down-regulation multiples were 0.48 and 0.58, respectively. Therefore, miR-15a and miR-92a were selected as key miRNAs for subsequent research.

### M2 macrophage-derived exosomes promote migration and invasion of glioma cells

**Fig. 2:**
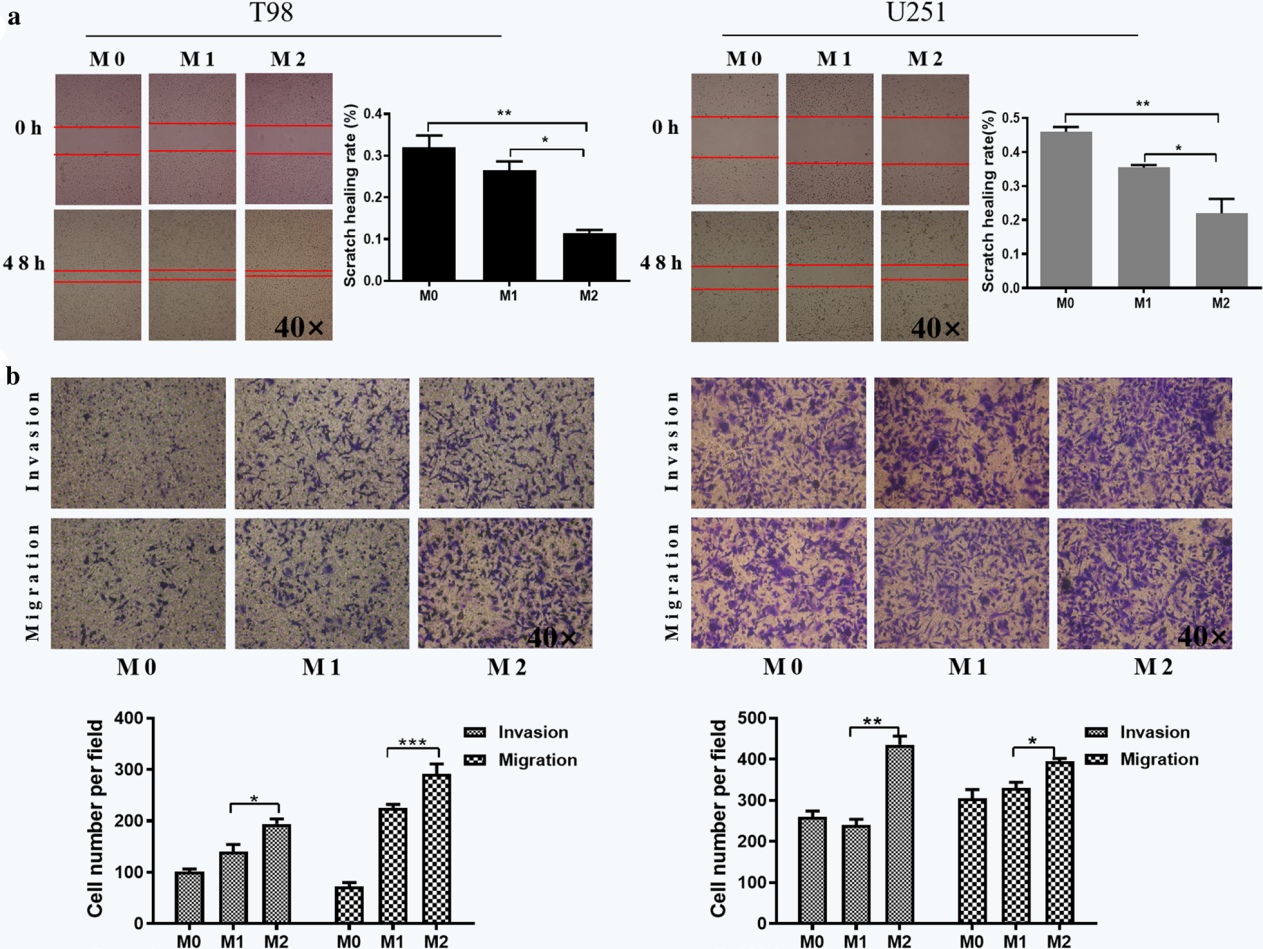
M2 macrophage-derived exosomes promoted migration and invasion of glioma cells. The effects of M2 macrophage-derived exosomes on **a** transverse migration, and **b** vertical migration and invasion in T98 and U251 cells as analyzed by scratch wound healing and transwell assays, respectively. Error bars denote standard deviation of triplicates. ***P < 0.001; **P < 0.01; *P < 0.05. Original magnification ×40 for scratch wound healing assays and transwell assays

With an aim to investigate the effect of exosomes derived from M2 macrophages on glioma cells, scratch wound healing and transwell assays were employed. We added the extracted exosomes of M0, M1 and M2 macrophages to glioma cells, respectively. The transverse migration ability of glioma cells was examined by scratch wound healing assays in T98 and U251 cell lines. It can be seen from Fig. 2a that the scratch healing rate of cells with M2 macrophage exosomes was faster than that of the M0 and M1 groups (P < 0.05). We obtained similar results in U251 cell line (Fig. 2a), indicating that exosomes derived from M2 macrophages can promote transverse migration of glioma cells.

The transwell assays were applied to investigate whether M2 macrophage exosomes could promote the vertical migration and invasion of glioma cells. We found that the number of migrating and invasive cells was significantly increased in M2 group compared with M0 and M1 groups in T98 cells (Fig. 2b). We obtained similar results in U251 cell line (Fig. 2b), suggesting that exosomes derived from M2 macrophages can promote vertical migration and invasion of glioma cells. Taken together, these findings elucidated that M2 macrophage exosomes can promote migration and invasion of glioma cells.

### Exosomal miR-15a and miR-92a inhibit migration and invasion of glioma cells

The foregoing results revealed that miR-15a and miR-92a were under-expressed in M2 macrophage exosomes and that exosomes could promote the migration and invasion of glioma cells. Therefore, we speculated that miR-15a and miR-92a might have an effect on glioma cells. After transfection of miR-15a and miR-92a into M2 macrophages, we collected cell culture supernatant and extracted exosomes. The results of qRT-PCR revealed that the expression of miR-15a was up-regulated about 4.9 times in M2 macrophage exosomes, and miR-92a was up-regulated about 7.0 times (Additional file 1: Figure S2), indicating that we successfully over-expressed miR-15a and miR-92a in M2 macrophage exosomes.

**Fig. 3:**
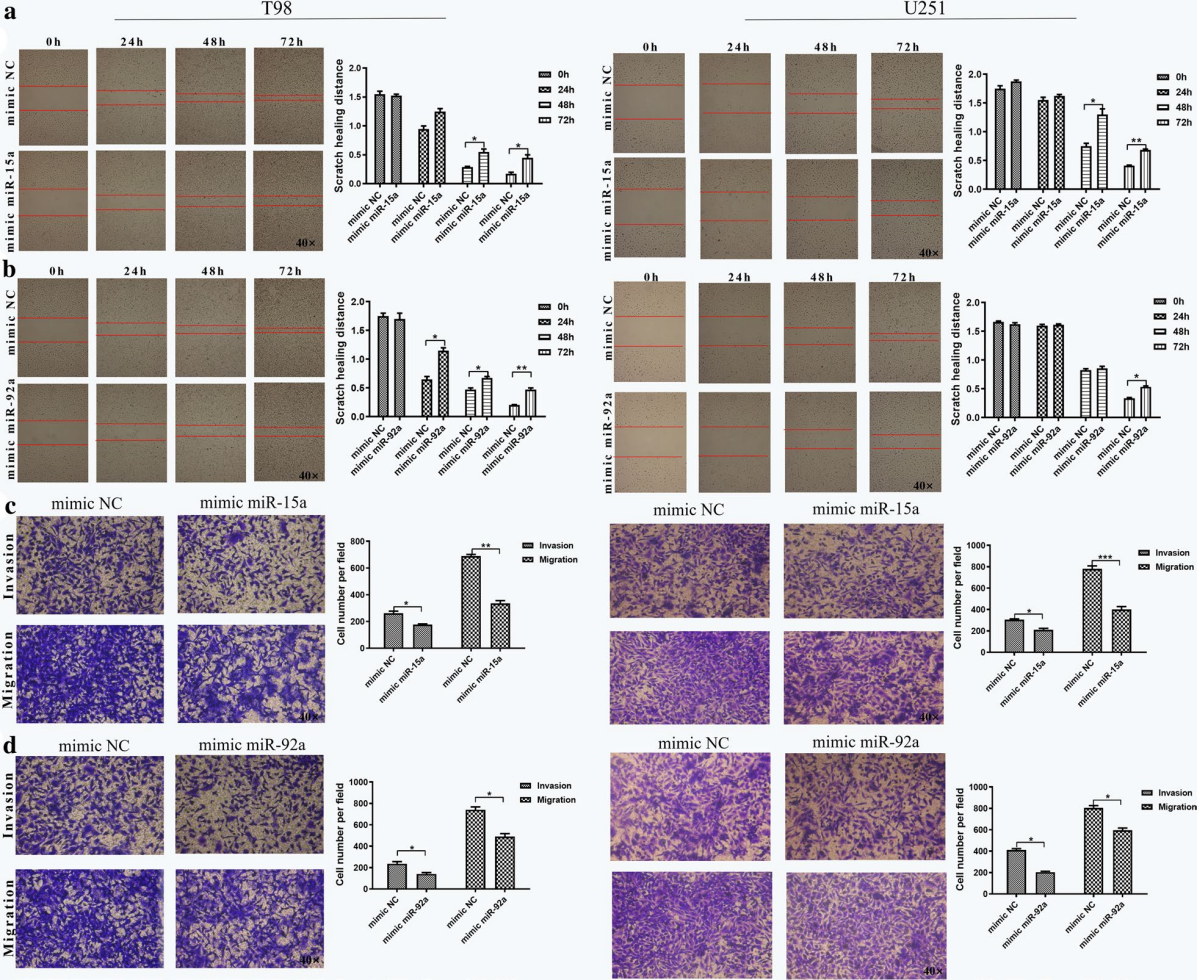
Exosomal miR-15a and miR-92a inhibited migration and invasion of glioma cells. The effects of exosomal miR-15a on **a** transverse migration, and **c** vertical migration and invasion in T98 and U251 cells and the effects of exosomal miR-92a on **b** transverse migration, and **d** vertical migration and invasion in T98 and U251 cells as analyzed by scratch wound healing and transwell assays, respectively. Error bars denote standard deviation of triplicates. ***P < 0.001; **P < 0.01; *P < 0.05. Original magnification ×40 for scratch wound healing assays and transwell assays

Subsequently, we reconnoitered the effects of exosomal miR-15a and miR-92a on the migration and invasion of glioma cells. We transfected miR-15a, miR-92a and mimic NC into M2 macrophages, collected cell culture supernatant 48 h later, extracted exosomes, and added them to T98 and U251 cell lines, respectively. The results from scratch would healing and transwell assays indicated that miR-15a over-expression inhibited the transverse migration of T98 and U251 cells (Fig. 3a, P < 0.05), as well as vertical migration and invasion (Fig. 3c, P < 0.05). We obtained similar results of miR-92a over-expression in T98 and U251 cell lines (Fig. 3b, d, P < 0.05). Collectively, exosomal miR-15a and miR-92a were found to inhibit migration and invasion of glioma cells.

### Target gene prediction

**Fig. 4:**
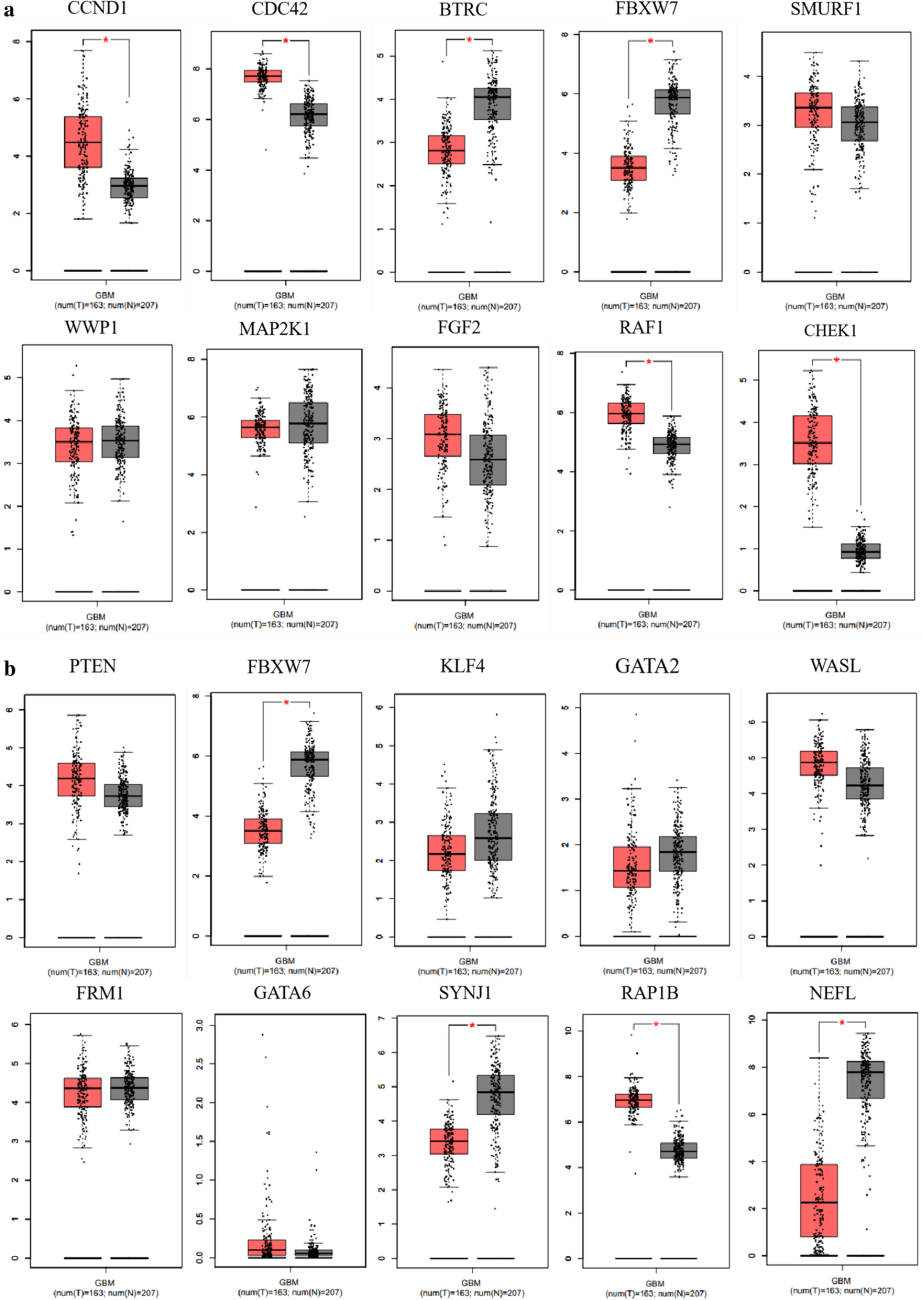
Expression of predicted hub gene targets of miR-15a and miR-92a in GEPIA. **a** Expression of ten hub genes for miR-15a and **b** miR-92a in glioma and adjacent tissues in GEPIA

To identify the target genes of miR-15a and miR-92a, picTar, miRanda, targetScan and PITA databases were used for prediction. A total of 304 target genes of miR-15a were obtained by intersecting the predicted target genes from the four online databases (Additional file 1: Figure S3A). Through STRING and Cytoscape, we found ten hub genes from the 304 target genes of miR-15a (Additional file 1: Figure S3B). Then we checked the expression of these ten hub genes in glioma and adjacent tissues in GEPIA. The results showed that CCND1, CDC42, RAF1, and CHEK1 were highly expressed in gliomas (Fig. 4a). Therefore, these four genes were selected for subsequent research. For miR-92a, we obtained 240 target genes (Additional file 1: Figure S3C), of which ten were identified as hub genes (Additional file 1: Figure S3D). According to the expression in glioma and adjacent tissues in GEPIA, only RAP1B was highly expressed in gliomas (Fig. 4b), which was chosen for following study.

### Target gene validation

In order to verify the target genes obtained from database prediction, we performed qRT-PCR, western blot and dual luciferase reporter gene assays in vitro. We transfected miR-15a, miR-92a and mimic NC into T98 and U251 cells, respectively. The qRT-PCR results showed that the over-expression efficiency of miR-15a in T98 and U251 cells was 11.67 and 8.4 times (Figure S4A), and that of miR-92a was 15.92 and 14.59 times (Additional file 1: Figure S4B), respectively. In addition, we verified the effect of target genes in cells through two different transfection methods of transfecting miR-15a and miR-92a into M2 type macrophages and collect exosomes or directly transfecting miR-15a and miR-92a into T98 cells. The results indicated that there was no evidently difference (Additional file 1: Figure S5).

**Fig. 5:**
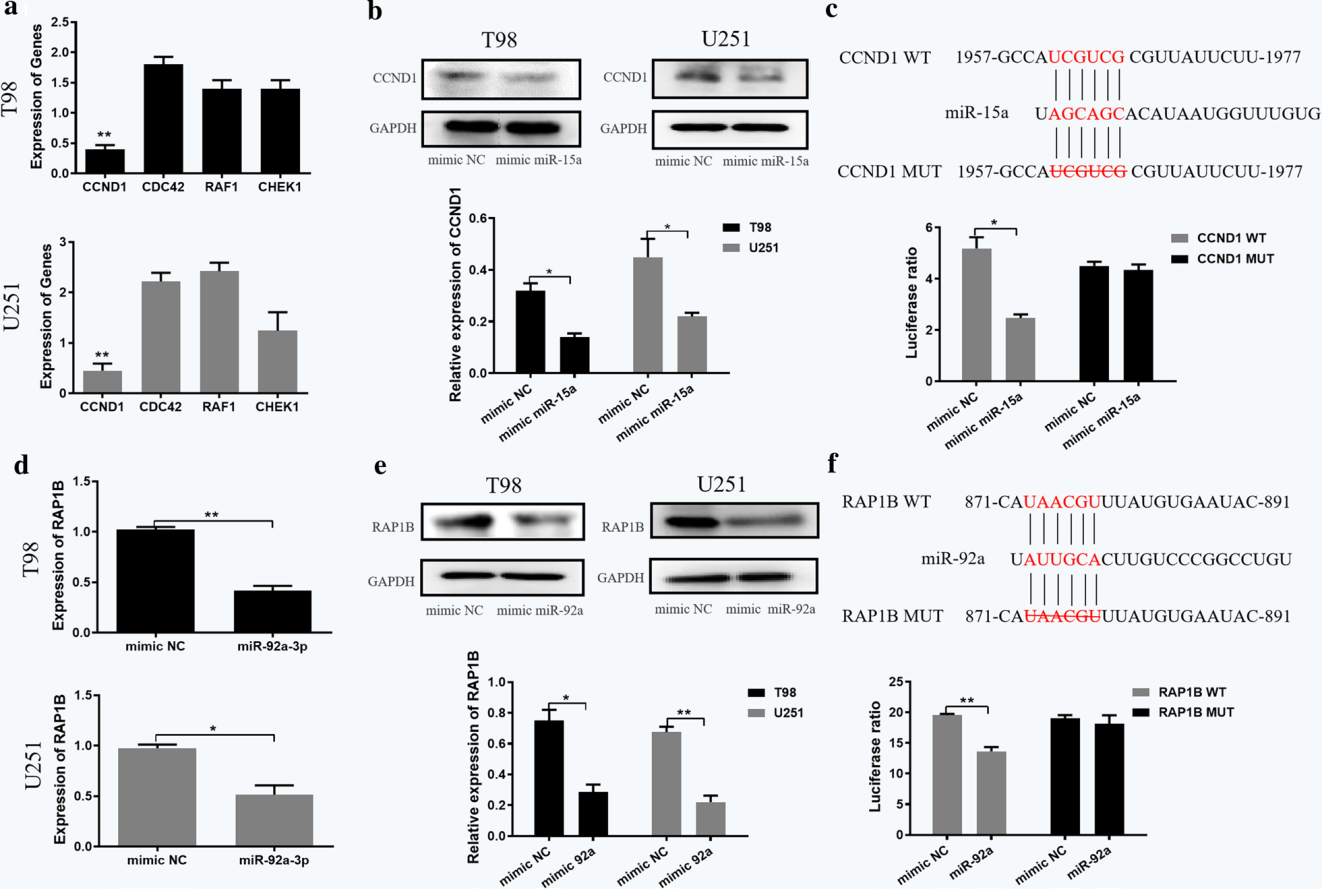
Target gene validation of miR-15a and miR-92a. Expression of selected target genes of **a** miR-15a and **d** miR-92a in T98 and U251 cell lines as analyzed by qRT-PCR assays. Protein expression of selected target genes of **b** miR-15a and **e** miR-92a in T98 and U251 cell lines as measured by western blot assays. Effects of **c** miR-15a and **f** miR-92a on the luciferase reporter activity of cells with CCND1 WT or CCND1 MUT and RAP1B WT or RAP1B MUT, respectively. Error bars denote standard deviation of triplicates. **P < 0.01; *P < 0.05. WT: wild type; MUT: mutant type

After the transfection of miR-15a, only CCND1 was down-regulated in T98 and U251 cell lines among the four predicted target genes (Fig. 5a). Western blot analysis also revealed that miR-15a over-expression could down-regulate the protein expression level of CCND1, so CCND1 was selected as the next research object (Fig. 5b). The relative luciferase activity was decreased in co-transfection of pGL3-CCND1-WT with miR-15a, compared with the control of mimic NC (p < 0.05), and there was no significant difference in luciferase activity in co-transfection of pGL3-CCND1-Mut with miR-15a (Fig. 5c), indicating that miR-15a was bound to the CCND1 gene. For miR-92a, qRT-PCR and western blot assays confirmed that miR-92a over-expression can down-regulate the gene and protein expression levels of RAP1B (Fig. 5d, e). In addition, the dual luciferase reporter gene assay confirmed that RAP1B was a target of miR-92a (Fig. 5f).

### RAP1B and CCND1 can activate the PI3K/AKT/mTOR signaling pathway

**Fig. 6:**
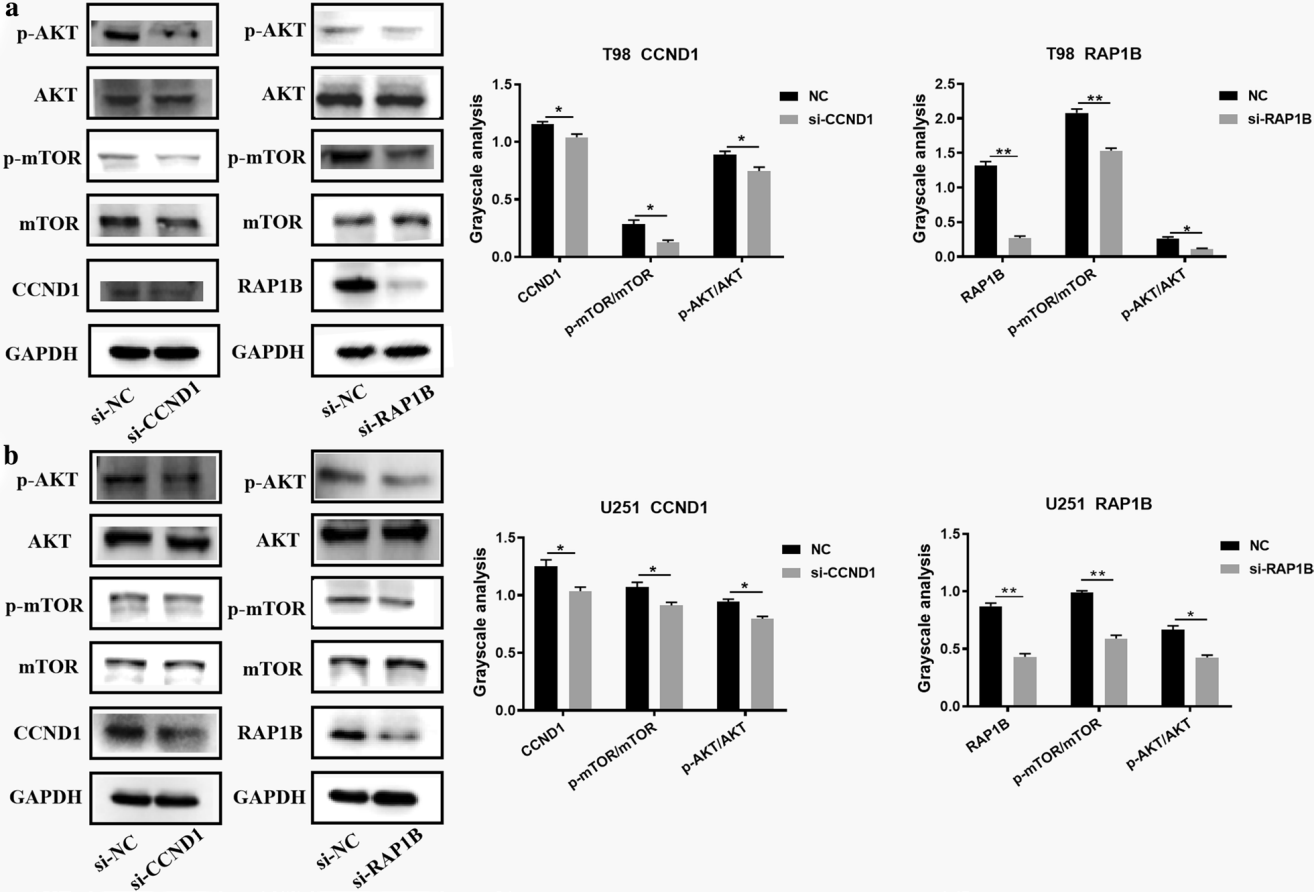
Knockdown of CCND1 or RAP1B inhibited the PI3K/AKT/mTOR signaling pathway. Western blot assays showed that the phosphorylation levels of AKT and mTOR were reduced compared with the control groups after interfering the expression of CCND1 or RAP1B in **a** T98 and **b** U251 cell lines. **P < 0.01; *P < 0.05

**Fig. 7:**
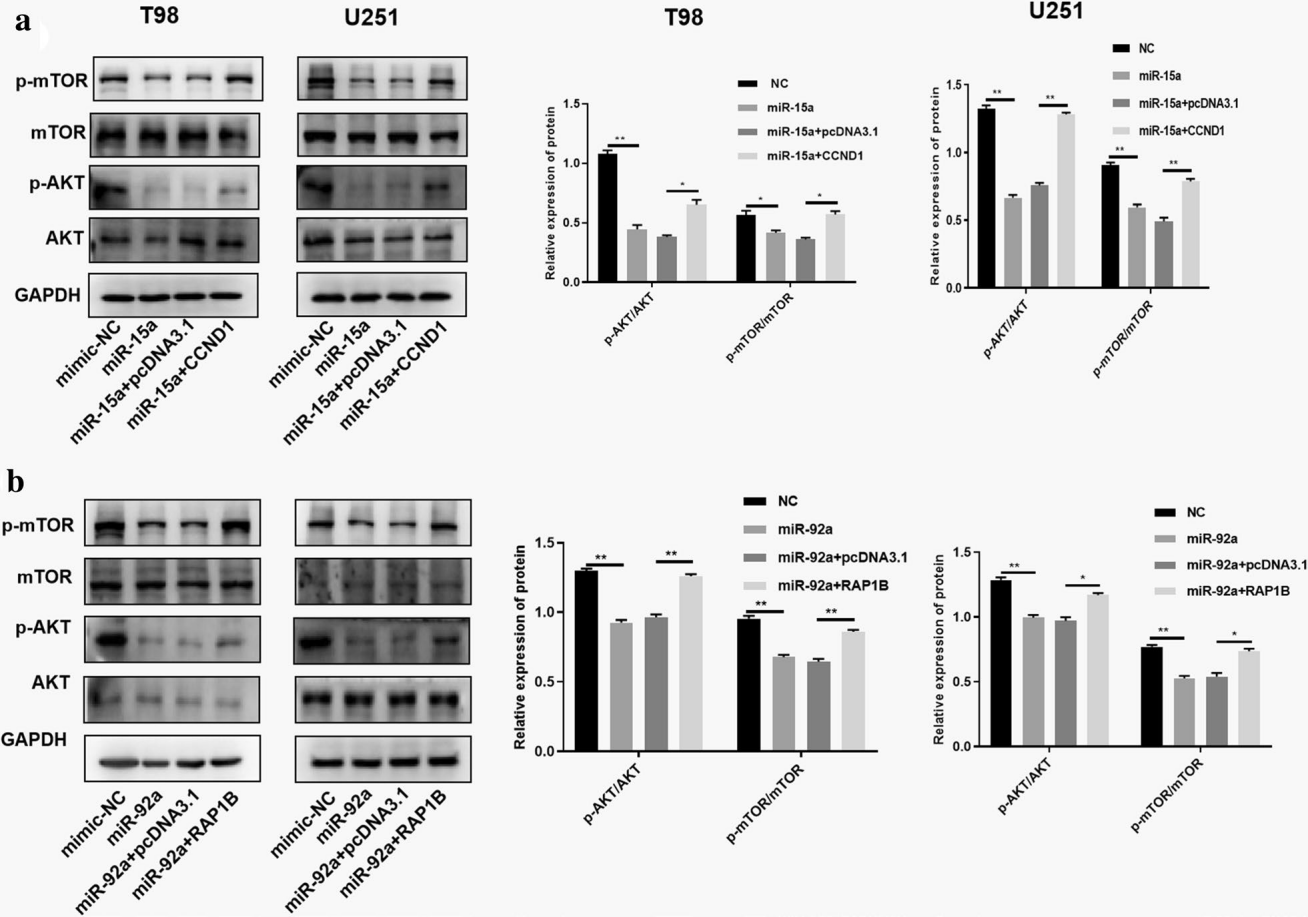
Effects of miR-15a and miR-92A on PI3K/AKT/mTOR signaling pathway. **a** miR-15a could block the PI3K/AKT/mTOR signaling pathway. The over-expression of CCND1 in the miR-15a group could reverse the blocking effect of miR-15a on the signaling pathway. **b** miR-92a could block the PI3K/AKT/mTOR signaling pathway. RAP1B in the miR-92a group could reverse the blocking effect of miR-92a on the signaling pathway

Studies have shown that PI3K/AKT/mTOR signaling pathway is closely related to the regulatory mechanism of glioma. We wondered whether CCND1 and RAP1B could affect the PI3K/AKT/mTOR signaling pathway in glioma. Therefore, si-CCND1 and si-RAP1B were transfected into T98 and U251 cell lines to study the effect of CCND1 and RAP1B on the PI3K/AKT/mTOR signaling pathway. As shown in Fig. 7, CCND1 knockdown in T98 and U251 cell lines reduced the phosphorylation level of AKT and mTOR compared to the NC group. For RAP1B, we obtained similar results by western blot analysis (Fig. 6a and b). Collectively, these results revealed that RAP1B and CCND1 could activate the PI3K/AKT/mTOR signaling pathway in glioma cells. To demonstrate whether macrophage-derived exosomal miR-15a and miR-92a have any effect on PI3K/AKT/mTOR signaling pathway in glioma cells. The relevant rescue experiments are performed. As shown in Fig. 7a, miR-15a could block the PI3K/AKT/mTOR signaling pathway. The expression levels of p-mTOR and p-AKT were down-regulated in the miR-15a group. The over-expression of CCND1 in the miR-15a group could reverse the blocking effect of miR-15a on the signaling pathway and rescue the protein levels of p-mTOR and p-AKT to a certain extent. Similarly, miR-92a could block the PI3K/AKT/mTOR signaling pathway. The expression levels of p-mTOR and p-AKT were down-regulated in the miR-92a group, and overexpression of RAP1B in the miR-92a group could reverse the blocking effect of miR-92a on the signaling pathway, and rescue the protein levels of p-mTOR and p-AKT to a certain extent (Fig. 7b).

## Discussion

Malignant glioma, the most common cancer of the central nervous system, accounts for around 70% of malignant primary brain tumors [19]. Patients with malignant glioma have a poor prognosis, with a median survival time of 3 years [20]. Hence, it is necessary to identify promising biomarkers to help diagnose gliomas early and to develop new therapeutic modalities with favorable prognosis. Exosomal miRNAs have reported to exert important physiological functions in tumorigenesis and development [17, 21]. In the present study, we identified seven differentially expressed miRNAs in infiltrating macrophages based on dataset GSE51332 from GEO database. Through qRT-PCR detection, miR-15a and miR-92a were found to be under-expressed in M2 macrophages, consistent with the expression levels in GEO. In addition, we confirmed the down-regulation of miR-15a and miR-92a in M2 macrophage exosomes. Subsequently, we demonstrated that M2 macrophage-derived exosomes promoted glioma migration and invasion in T98 and U251 cell lines, whereas exosomal miR-15a and miR-92a inhibited the migration and invasion of glioma cells. We thus speculated that miR-15a and miR-92a might inhibit the invasion and migration of gliomas.

According to bioinformatics analysis, we identified four target genes for miR-15a and one for miR-92a. We then selected CCND1 as the key target gene for miR-15a since only CCND1 was down-regulated at the transcript and protein levels after miR-15a was over-expressed. Similarly, RT-qPCR and western blot assays confirmed that miR-92a over-expression was associated with significant reduction in levels of RAP1B transcript and protein. Through dual luciferase reporter gene assays, we verified that CCND1 and RAP1B are the target genes of miR-15a and miR-92a, respectively.

CCND1, namely G1/S-specific cyclin-D1, regulates the cell cycle process during the transition from G1 to S phase. Increasing studies show that many genes affect the viability and migration of glioma cells by regulating CCND1. For example, Alqudah MA elucidated that via activation of CCND1 and EGFR, NOTCH3 promotes glioma cell proliferation, migration and invasion [22]. Similar to our results, the reduction of miR-17 in glioma cells improves cell viability and migration ability by increasing the expression of CCND1, p-Akt and Akt [23].

RAP1B encodes a member of the RAS-like small GTP-binding protein superfamily, which regulates a variety of cellular processes, including cell adhesion, growth, and differentiation [24]. Accumulating data indicate that the deregulated activation of RAP1B is related to a series of malignant tumors, and that RAP1B has effects on cell proliferation, metastasis, angiogenesis, and treatment resistance [25, 26]. In addition, numerous studies have reported that CCND1/RAP1B is associated with glioma cell proliferation and invasion [27–30]. Consistent with our findings, studies have shown that abnormal miRNA expression can affect gliomas by targeting RAP1B. For example, She et al. demonstrated that overexpression of miR-181 can inhibit the aggressive proliferation of glioblastoma cells by targeting RAP1B-mediated cytoskeletal remodeling and related molecular changes [31].

Furthermore, we demonstrated that knockdown of CCND1 or RAP1B could inhibit the PI3K/AKT/mTOR signaling pathway. Moreover, in the rescue experiment, we found that miR-15a and miR-92a could block the PI3K/AKT/mTOR signaling pathway. Overexpression of CCND1/RAP1B in miR-15a/miR-92a group can reverse the blocking effect of miR-15a/miR-92a on the signaling pathway, and rescue the phosphorylated protein in a certain extent. It has been demonstrated that many microRNAs as biomarkers for glioblastoma [32–34]. However, recent evidence suggests that microRNAs could migrate between cells and mediate repression of target genes [35]. In addition, miRNAs play a negative regulation and confer characteristic changes in the expression levels of target genes, and may regulate a variety of signaling pathways, and will generate integral effects on recipient cells [36]. To some extent, the above research provides new insights into the mechanism research. p-AKT and p-mTOR were reported to be activated or over-expressed in human gliomas, and the labeling index of the PI3K/AKT/mTOR pathway increased with increasing grade of malignancy [37, 38]. In gliomas, the PI3K/AKT/mTOR pathway helps to induce invasion and angiogenesis in cells, and patients with activated PI3K/AKT/mTOR pathway have a worse prognosis than those without carcinogenic activation of the pathway [39, 40]. In addition, BRCA1-associated proteins inhibit glioma cell proliferation and migration through the TGF- AKT/PI3K/mTOR signaling pathway [41] To some extent, the above research [13]. Besides in gliomas, this pathway was involved in the pathological mechanism of other cancer. For example, Xu et al. reported that exosome MALAT1 promotes the malignant behavior of CRC cells by activating the PI3K/Akt/mTOR pathway [42]. Similarly, the other article reported that Dhw-208 inhibits the growth of human breast cancer cells by inhibiting the PI3K/AKT/MTOR signaling pathway [43]. BRCA1-associated proteins inhibit glioma cell proliferation and migration through the TGF-AKT/PI3K/mTOR signaling pathway [13].

**Fig. 8:**
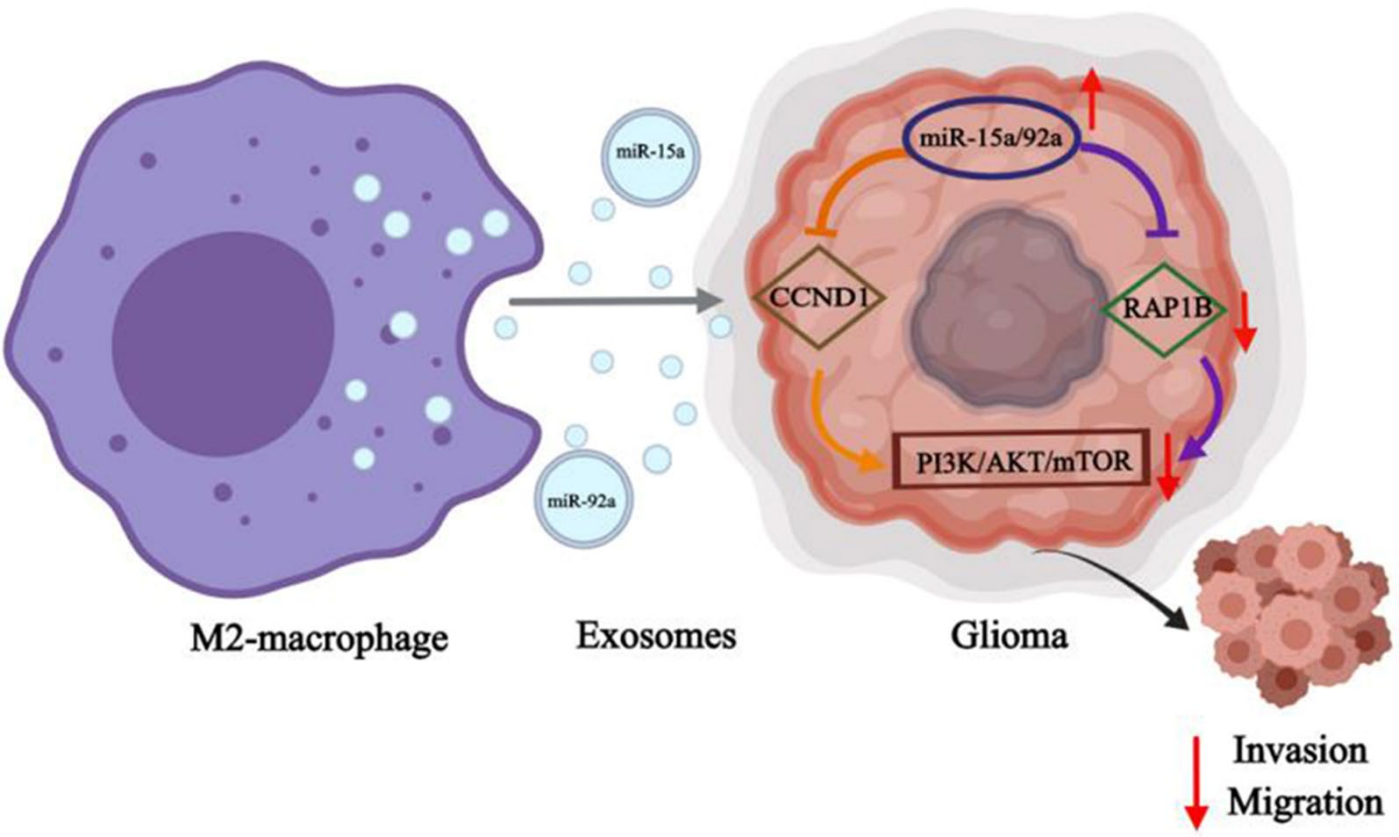
Mechanism diagram of miR-15a and miR-92a in gliomas

To better understanding our study, we deduced a mechanism diagram of miR-15a and miR-92a (Fig. 8): M2 macrophages secrete miR-15a and miR-92a to glioma cells through exosomes, and then miR-15a and miR-92a separately bind to CCND1 and RAP1B, thereby blocking the PI3K/AKT/mTOR signaling pathway to inhibit glioma invasion and migration.

As is well known to all, the main function of M2-type macrophages is to promote the progress of tumor cells. For the first time, our findings elucidated that M2 macrophage-derived miR-15a and miR-92a could be secreted through exosomes to inhibit the invasion and migration of glioma cells. Combining bioinformatics analysis and experiments, CCND1 and RAP1B were found to be the target genes of miR-15a and miR-92a, respectively. To sum up, we unearthed that M2 macrophage-derived miR-15a and miR-92a could target CCND1 and RAP1B, respectively, thereby inhibiting the invasion and migration via blocking PI3k/AKT/mTOR signaling pathway in gliomas. Despite these promising results, the limitations cannot be ignored. In this paper, there is a lack of animal experiments and some rescue experiments for verification. In addition, whether miR-15a and miR-92a can affect the differentiation of macrophages. M1 macrophages mainly play an anti-tumor and pro-inflammatory role, while M2 macrophages chiefly promote tumor progression. If miR-15a and miR-92a can affect the proportion of these two phenotypes of macrophages, they will also play a role in glioma invasion and migration, which will be our future research direction.

## Methods

### Cell lines

Glioma cell lines (T98 and U251) and human mononuclear macrophage line (THP-1) were purchased from Cell Bank of Chinese Academy of Sciences (http://www.cellbank.org.cn/). T98 and U251 cells were incubated in Dulbecco’s modified Eagle’s medium (DMEM, 31600034, Hyclone, Logan, UT, USA) supplemented with 10% fetal bovine serum (FBS, 10099141, Gibco, Grand Island, NY, USA). THP-1 cells were treated with 100 ng/mL phorbol 12-myristate 13-acetate (PMA, P1585, Sigma, St.Louis, Missouri, USA) for 24 h for differentiation into macrophages. Next, cells were treated with 100 ng/mL lipopolysaccharide (LPS, P8139, Sigma, St.Louis, Missouri, USA) and 20 ng/mL interferon-γ (IFN-γ, 285-IF, R&D, Minneapolis, MN, USA) for 24 h, polarizing them into M1 phenotype. After treatment with 20 ng/mL interleukin 4 (IL-4, AF-200-04-5, Peprotech, Rocky Hill, NJ, USA) for 72 h, the cells were polarized to M2 phenotype.

### Cell treatment and grouping

THP-1 was considered as a control group without any transfection, while Exo was considered as an experimental group with M2 macrophage exosomes. Once the cell fusion in T98 and U251 cells reached 80–90%, transfection was performed according to the instructions provided by Lipofectamine 2000 (11668-019, Invitrogen, New York, CA, USA). The cells were grouped into mimic NC (transfection of mimic NC), mimic miR-15a (transfection of miR-15a), and mimic miR-92a (transfection of miR-92a).

### Isolation and characterization of exosomes

Once the confluence of macrophages reached 80–90%, the complete medium was discarded and replaced with fresh medium. Next, we collected 30 mL of used cell culture medium in each cell line and isolated the exosomes by centrifugation at 4° C. After additional centrifugation at 4 °C at 100,000*g* overnight, the exosomes were extracted in accordance with the instructions on the ExoQuick (System Bioscience, Mountain View, CA, USA). The precipitation was then washed with a large amount of PBS, resuspended in PBS and stored at − 80 °C for further use. The exosomal suspension was concentrated and the concentration of exosomes was measured using the bicinchoninic acid (BCA) kit (23227, Thermo Fisher Scientific, Waltham, MA, USA).

Subsequently, exosomes were identified using JEM-2010 HT transmission electron microscope. 20 μL of exosomes was added dropwise to a copper mesh and left for 3 min. Then filter paper was used to suck up the liquid from the sides. Next, 30 μL of phosphotungstic acid solution (pH 6.8) was added to counter-staine exosomes for 5 min at room temperature. After baking, exosomes were photographed under the transmission electron microscope. Western blot analysis was then used to identify exosomal surface markers, such as tumor susceptibility genes 101 (TSG101), CD63, and CD9.

### Transmission electron microscopy (TEM) and Nanoparticle-trancking analysis (NTA) analysis

NTA and TEM were used to identify exosomes from macrophage cell. Exosome sample were added to copper grids at room temperature for 5 min, stained using2% uranyl acetate solution for 1 min, dried for 20 min at room temperature, and then observed using TEM (JEOL, Ltd.). Besides, the size distribution of the exosomes was measured with NanosizerTM technology (Malvern Instruments, Malvern, UK), and was analysed using Zetasizer software (Malvern).

### Reverse transcription quantitative polymerase chain reaction (RT-qPCR)

Total RNA was extracted by Trizol reagent (Invitrogen, Carlsbad, CA) according to the manufacturer’s instructions. Reverse transcription was carried out after total RNA was extracted, according to instructions of UEIris II RT-PCR System for First-Strand cDNA Synthesis (US Everbright^®^Inc, Suzhou, China). SYBR Premix Ex Taq (US Everbright^®^Inc, Suzhou, China) on an ABI 7900 system (Applied Biosystems, Foster City, CA, USA) was employed to conduct qRT-PCR assay using glyceraldehyde 3‐phosphate dehydrogenase (GAPDH) as endogenous controls. The 2 − ΔΔCt method was employed to detect the comparative quantification. The primers were purchased from Sangon Biotech (Shanghai, China) and the sequences were shown in Table 2.

**Table 2:** List of primers used in this study

Name	F Sequences (5′-3′)	R Sequences (5′-3′)
CD206	ATGTTGAAGGGACGTGGCTG	TCCGTTCACCAGAGGGATCT
IL-10	GACTTTAAGGGTTACCTGGGTTG	TCACATGCGCCTTGATGTCTG
TNF-a	AGGCACTCCCCCAAAAGATG	TGGTGGTTTGTGAGTGTGAGG
IL-12	TTGAGGTCATGGTGGATGCC	GGTCAGGTTTGATGATGTCCCT
CCND1	ACAGCGTGAGAGGTACTAGGT	CTTGGGGTCCATGTTCTGCT
RAP1B	ACAGCGTGAGAGGTACTAGGT	GTAAATTGCTCCGTTCCTGC
GAPDH	GCTCTCTGCTCCTCCTGTTC	CGACCAAATCCGTTGACTCC
CDC24	CGACCGCTGAGTTATCCACA	TCTCAGGCACCCACTTTTCT
RAF1	GATGCCGTGTTTGATGGCTC	CCATTTCGCACATTGACCACT
CHEK1	ATGAAGCGTGCCGTAGACTG	TGCAGATAAACCACCCCTGC

F: forward; R: reverse

### Scratch wound healing assays

The scratch wound healing assay was used to evaluate the transverse migratory ability of cells. Briefly, 1 × 106/mL cells were plated on 6-well plates and scraped by a 10 µl tip to generate uniform wounds. The wells were washed three times with phosphate-buffered saline (PBS) to remove the exfoliated cells, and then medium with 2% fetal bovine serum was added. The cells were further incubated in a 37 °C and 5% CO_2_ incubator. The photos at a magnification of 40× were taken at 0, 24, 48 and 72 h, respectively.

### Transwell assays

Transwell chambers uncoated or coated with Matrigel (BD Biosciences, USA) were employed to assess cell vertical migratory or invasive ability, respectively. In brief, the cells were digested to a concentration of 1 × 105 cells/ml and seeded in the upper chamber with serum-free medium. At the same time, 600 µl of 10% FBS-DMEM was added to the lower layer. Then exosomes derived from M0/M1/M2 macrophages and M2 macrophages transfected with mimic NC/miR-15a/miR-92a were added to the culture medium in the lower chamber, respectively. After 24 h, the upper device was secured and washed by PBS carefully and then fixed with 4% polyformaldehyde for 20 min at room temperature. Then the chamber was stained in a 24-well plate containing 600 μL of crystal violet for 20 min. The inside of chamber was carefully wiped with medical cotton. The cells were then moved to a new 24-well plate, and observed under a microscope at a magnification of 40×.

### Western blot analysis

Total protein of cell or exosome was extracted after 48 h transfection using cell lysis buffer (Beyotime Biotechnology Shanghai, China). The supernatant was collected after centrifugation (12,000 rpm) at 4 °C for 15 min. Protein samples were separated using 12% sodium dodecyl sulfate polyacrylamide gel electrophoresis (SDS-PAGE) after boiling for 10 min with loading buffer (Beyotime Biotechnology Shanghai, China), and transferred to a polyvinylidene fluoride (PVDF) membrane at 200 mA for 120 min. After blockade by 5% BSA at room temperature for 60 min, blots were probed with diluted primary antibodies: anti-CD206 (ab64693, 1:1000), CD68 (ab125212, 1:1000), CD9 (ab223052, 1:1000), CD63 (ab216130, 1:1000), TSG101 (ab30871, 1:1000), CCND1 (ab226977, 1:1000), RAP1B (ab154756,1:1000), p-AKT (ab38449, 1:1000), AKT (ab18785, 1:1000), p-mTOR (ab109268, 1:1000), mTOR (ab2732, 1:1000) and glyceraldehyde-3-phosphate dehydrogenase (GAPDH) (ab8245, 1:5000) at 4 °C overnight. Subsequently, the membrane was incubated with horseradish peroxidase (HRP)-labeled goat anti-rabbit IgG (ab205718, 1:20000) or goat anti-mouse (ab6789, 1:5000) dilution for 1 h at room temperature. Antibodies were all purchased from Abcam Inc. (Cambridge, UK). The images of the gels were scanned using Bio-Rad Gel Doc XR + system (Bio-Rad, Hercules, CA, USA). GAPDH was used as an internal control. The protein levels were expressed as the ratio of target bands to GAPDH.

### Bioinformatics analysis

We retrieved the gene expression data related to gliomas from GEO database (https://www.ncbi.nlm.nih.gov/geo/) and then performed differential expression analysis with screening conditions of │log2FC│ > 1 and *P *< 0.05 by GEO2R (http://www.ncbi.nlm.nih.gov/geo/geo2r/). Subsequently, the potential target genes of miR-15a and miR-92a were predicted using TargetScan [44], picTar [45], miRanda [46] and PITA [47] databases. Next, we used STRING [48] database (https://string-db.org/) to make the protein–protein interactions of the selected target genes and imported them into Cytoscape [49] to obtain hub genes. The expression of the top 10 hub genes was detected via GEPIA database (http://gepia.cancer-pku.cn/index.html).

### Dual luciferase reporter gene assays

Targeting relationships of CCND1 and miR-15a, RAP1B and miR-92a were confirmed by the Dual luciferase reporter gene assays, respectively. The wild type (WT) containing the predicted target site, and the mutant type (MUT) with the binding site deleted were amplified and cloned into the pGL3 plasmid to generate pGL3-CCND1-WT and pGL3-CCND1-MUT, pGL3-RAP1B-WT and pGL3-RAP1B-MUT vectors. Subsequently, the luciferase vectors were transfected into HEK293T cells together with miR-15a, miR-92a or mimic NC, respectively. After 24 h transfection, the relative luciferase activity was measured by normalizing the firefly luminescence to the Renilla luminescence using the Dual-Luciferase Reporter Assay System (Promega, Madison, WI, USA) following manufacturer’s protocol.

### Statistical analysis

All statistical analyses were carried out using GraphPad Prism 7.0 (La Jolla, CA, USA). Differences between two dependent groups were assessed by the Student *t* test. As for multiple groups, the comparisons were performed with one-way analysis of variance. P < 0.05 was considered to be statistically significant. All data in this work are presented as the mean ± SD.

## Conclusion

Taken together, the research results in this paper indicated that the exosomes miR-15a and miR-92a derived from M2 macrophages inhibit the migration and invasion of glioma cells through the PI3K/AKT/mTOR signaling pathway (Additional file 2).

## Supplementary information

**Additional file 1.**

**Additional file 2.**

## Data Availability

The data supporting the findings of this study can be obtained from the corresponding author according to the reasonable request.

## Abbreviations

TAM: Tumor-associated macrophages
miRNAs: microRNAs
miR-15a: hsa-miR-15a-5p
miR-92a: hsa-miR-92a-3p
qRT-PCR: Quantitative real-time polymerase chain reaction
GAPDH: Glyceraldehyde 3‐phosphate dehydrogenase
PBS: Phosphate-buffered saline
SDS-PAGE: Sodium dodecyl sulfate polyacrylamide gel electrophoresis
PVDF: Polyvinylidene fluoride
HRP: Horseradish peroxidase
MUT: Mutant type
