# Correction to: M2 macrophage-derived exosomal microRNAs inhibit cell migration and invasion in gliomas through PI3K/AKT/mTOR signaling pathway

**DOI:** 10.1186/s12967-021-02920-4

**Published:** 2021-06-16

**Authors:** Jie Yao, Zefen Wang, Yong Cheng, Chao Ma, Yahua Zhong, Yilei Xiao, Xu Gao, Zhiqiang Li

**Affiliations:** 1 Human Genetic Resources Conservation Center of Hubei Province, Wuhan, 430071 China; 2 Tumor Precision Diagnosis and Treatment Technology and Translation Medicine, Hubei Engineering Research Center, Wuhan, 430071 China; 3 Department of Physiology, Wuhan University School of Basic Medical Sciences, Wuhan, 430071 China; 4 Department of Neurology, Hankou Hospital, General Hospital of Central Theater Command of Chinese People’s Liberation Army, Wuhan, 430014 China; 5 Department of Neurosurgery, Zhongnan Hospital of Wuhan University, No 169 Donghu Road, Wuhan, 430071 Hubei China; 6 Department of Oncology, Zhongnan Hospital of Wuhan University, Wuhan, 430071 China; 7 Department of Neurosurgery, Liaocheng People’s Hospital, Liaocheng, 252000 China; 8 Department of Neurosurgery, General Hospital of Northern Theater Command of People’s Liberation Army, Shenyang, 110000 China

## Correction to: J Transl Med (2021) 19: 99 https://doi.org/10.1186/s12967-021-02766-w

In the original publication [1] there was an error in Fig. 3. In the experiments every sample was shot from many different visual fields. However, due to human error a wrong figure with the same group of different visual field was uploaded.

In this correction article the correct (Fig. 1) and incorrect (Fig. 2) version of Fig. 3 are published. The full captions are available via the original article. The original article has been updated.

**Fig. 1:**
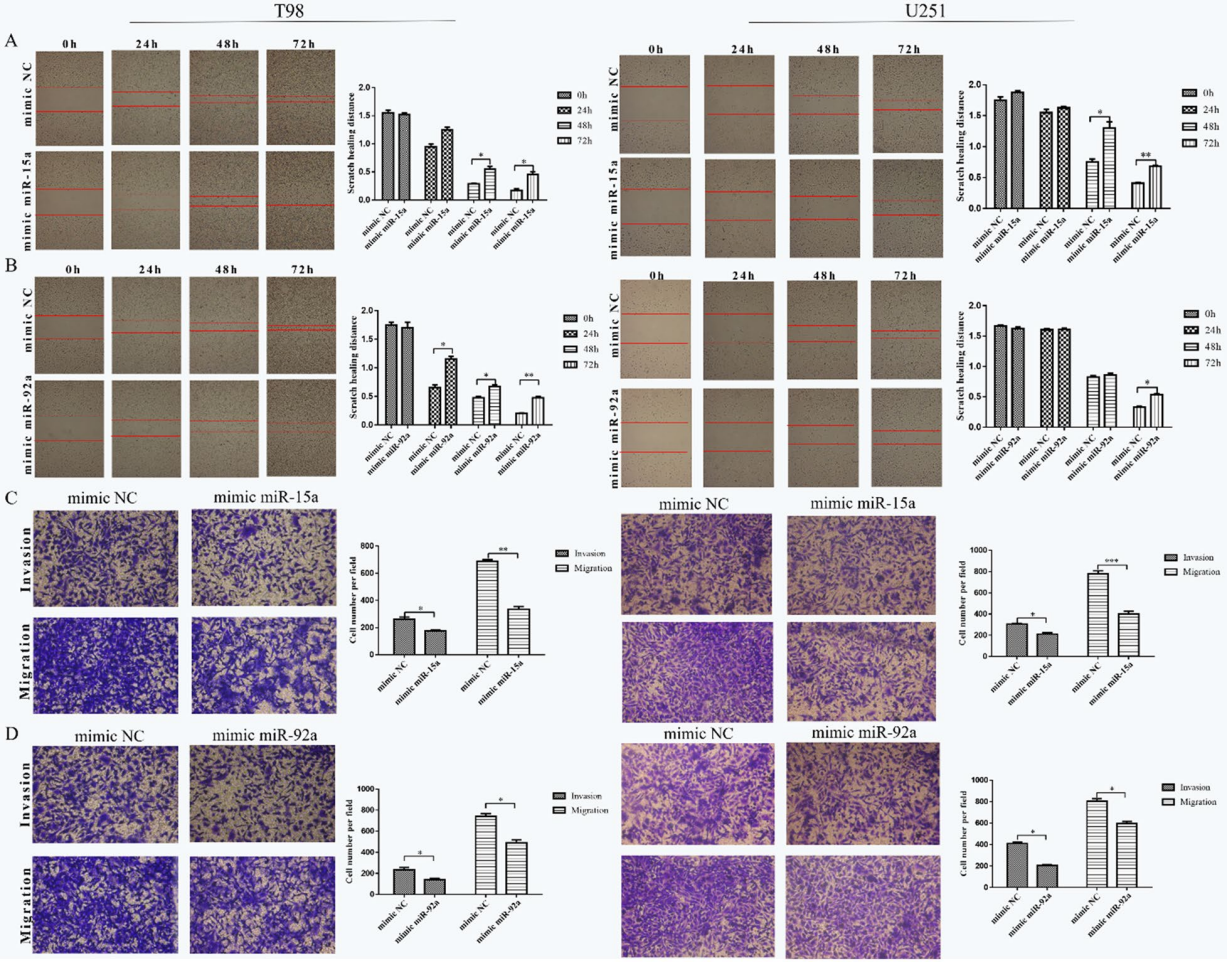
The corrected Fig. 3

**Fig. 2:**
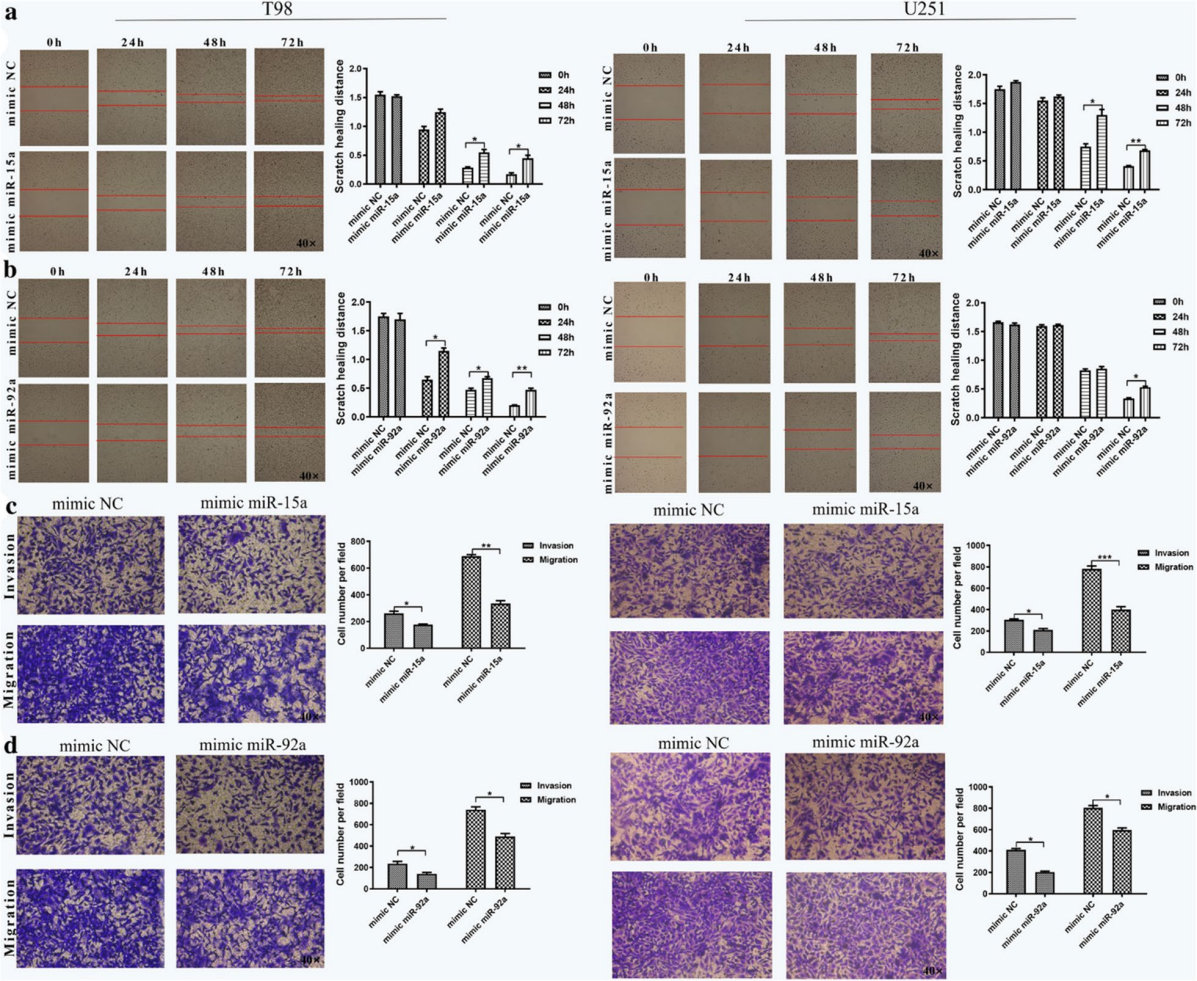
Incorrect Fig. 3 as originally published
