# Supplementary material for: M2 macrophage-derived exosomal microRNAs inhibit cell migration and invasion in gliomas through PI3K/AKT/mTOR signaling pathway
Source: J Transl Med. 2021 Mar 6;19:99. doi: 10.1186/s12967-021-02766-w (PMC7937290; doi:10.1186/s12967-021-02766-w)
Supplement: Supplementary file 1 — Additional file 1. [file 12967_2021_2766_MOESM1_ESM.docx]

**Supplemental figures**

**
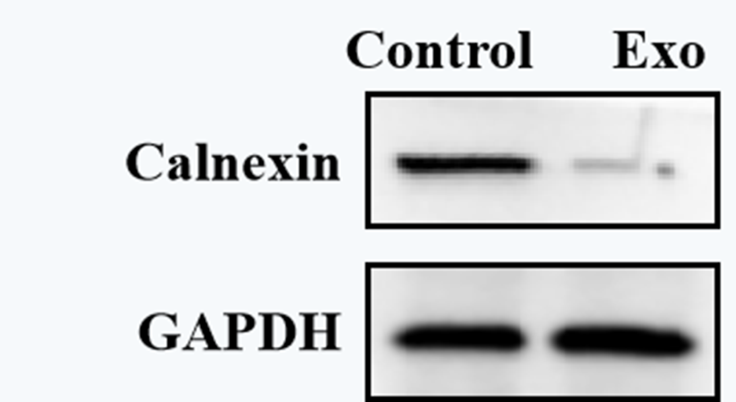
**

**Supplemental Figure S1 The Calnexin protein expression level was measured by a western blot assay**


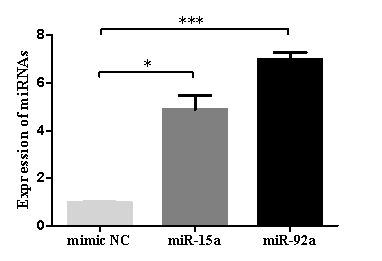


**Supplemental Figure S2.** Over-expression efficiency of miR-15a and miR-92a in M2 macrophages.


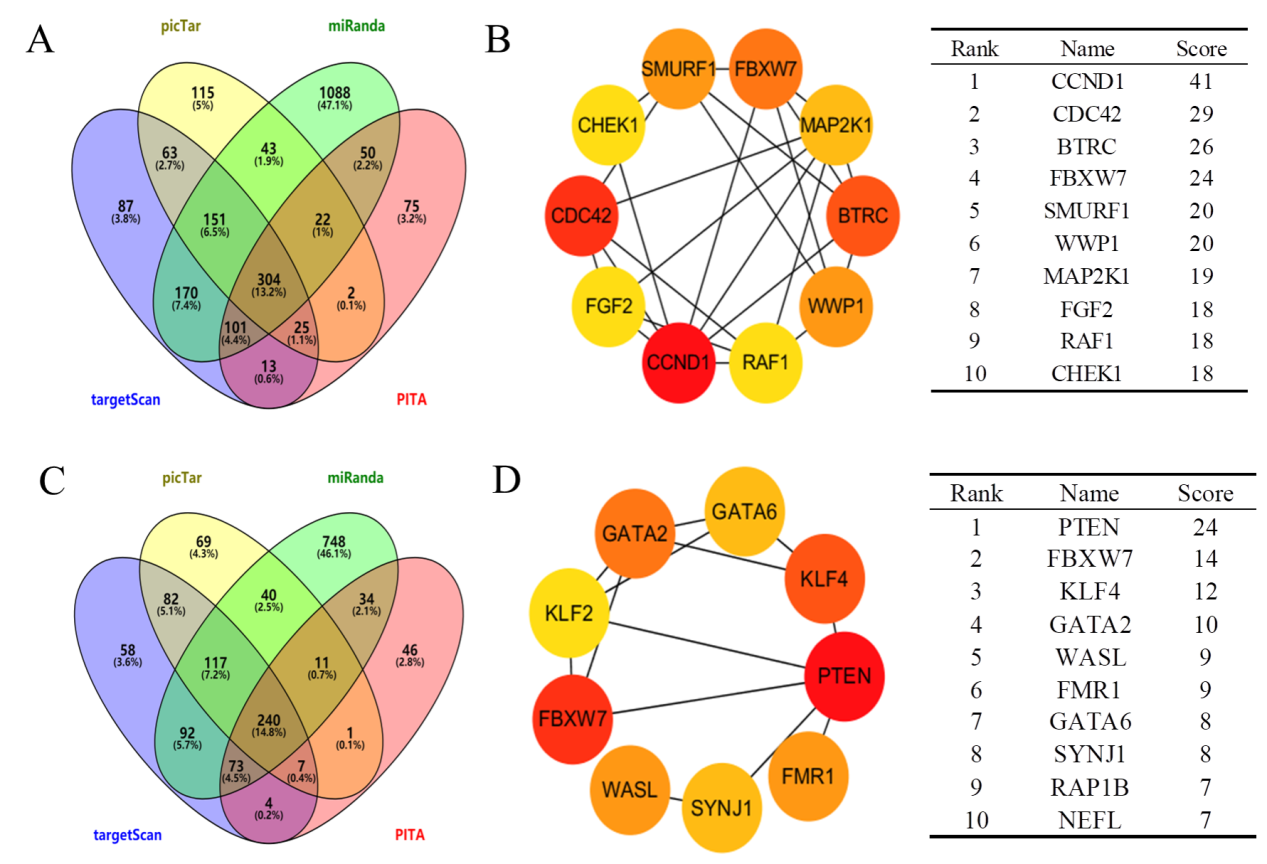


**Supplemental Figure S3. Target gene prediction of miR-15a and miR-92a by bioinformatics analyses. (A)** Venn's diagram of target genes of miR-15a predicted by four databases. **(B)** Interaction between ten hub genes for miR-15a by Cytoscape (left panel) and genes ranked according to score (right panel). (**C**) Venn's diagram of target genes of miR-92a predicted by four databases. (**D**) Interaction between ten hub genes for miR-92a by Cytoscape (left panel) and genes ranked according to score (right panel).

**
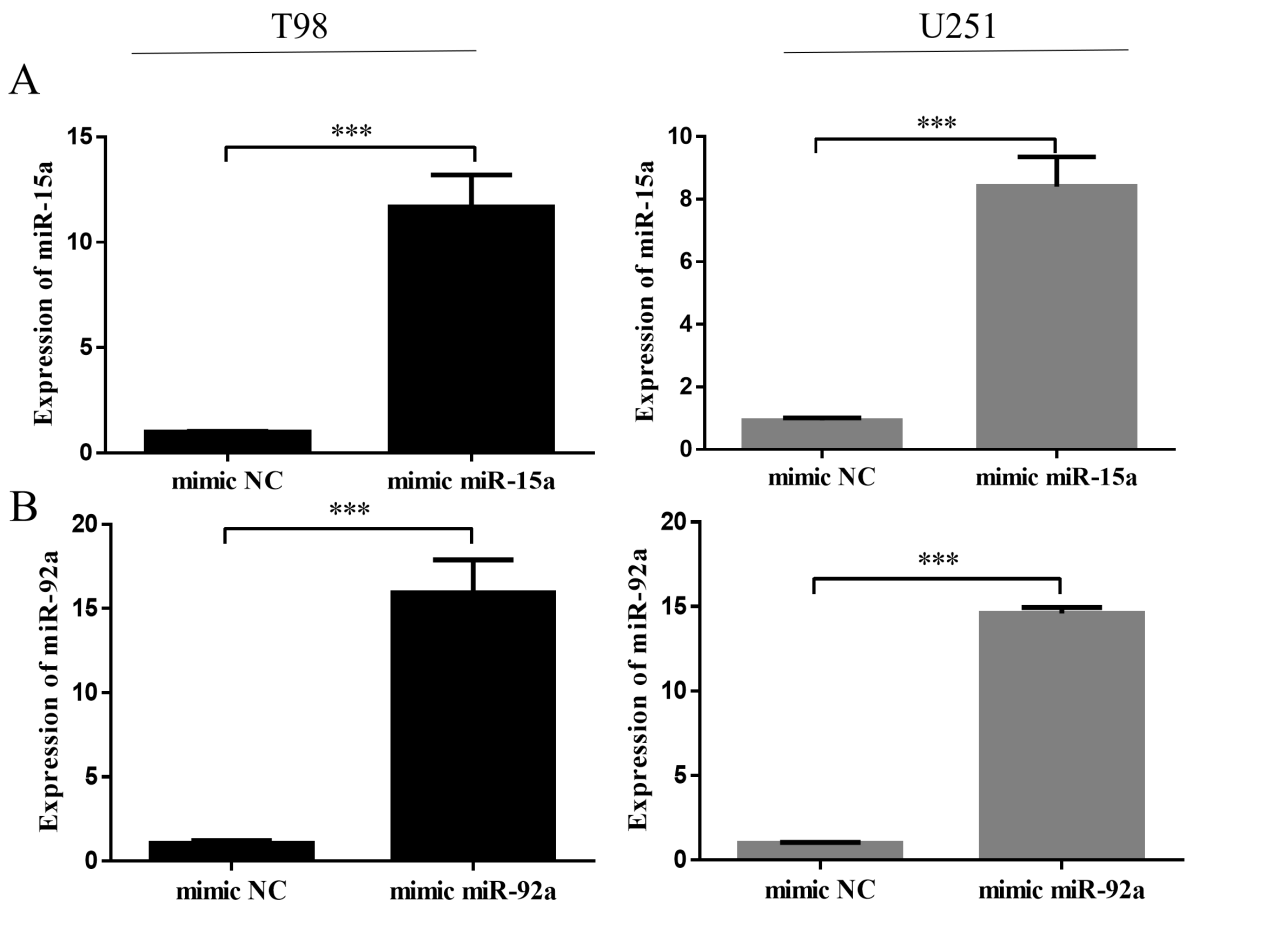
**

**Supplemental Figure S4.** Over-expression efficiency of (A) miR-15a and (B) miR-92a in T98 and U251 cell lines.

**
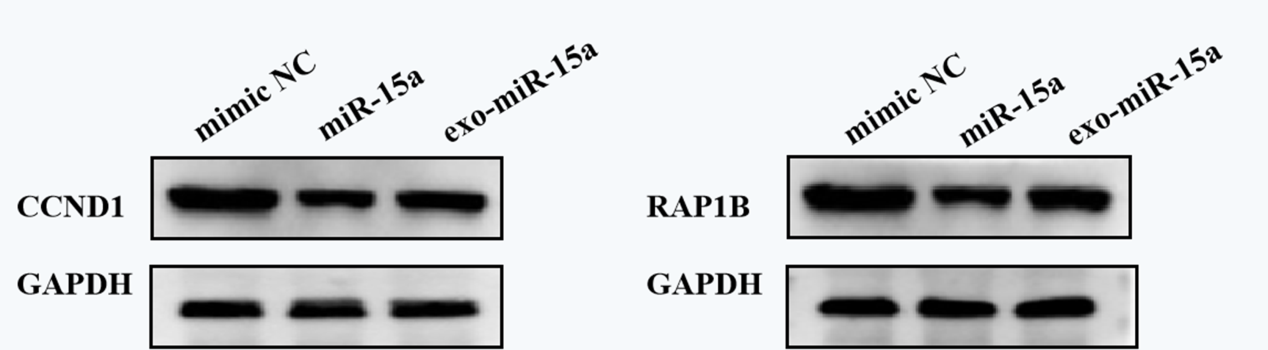
**

**Supplemental Figure S5** The protein expression of miR-15a and miR-92a target genes was verified by western blot assay.
